# Analysis of the Replication Mechanisms of the Human Papillomavirus Genomes

**DOI:** 10.3389/fmicb.2021.738125

**Published:** 2021-10-18

**Authors:** Lisett Liblekas, Alla Piirsoo, Annika Laanemets, Eva-Maria Tombak, Airiin Laaneväli, Ene Ustav, Mart Ustav, Marko Piirsoo

**Affiliations:** Institute of Technology, University of Tartu, Tartu, Estonia

**Keywords:** HPV, virus replication, non-theta replication, 2D, theta replication

## Abstract

The life-cycle of human papillomaviruses (HPVs) includes three distinct phases of the viral genome replication. First, the viral genome is amplified in the infected cells, and this amplification is often accompanied by the oligomerization of the viral genomes. Second stage includes the replication of viral genomes in concert with the host cell genome. The viral genome is further amplified during the third stage of the viral-life cycle, which takes place only in the differentiated keratinocytes. We have previously shown that the HPV18 genomes utilize at least two distinct replication mechanisms during the initial amplification. One of these mechanisms is a well-described bidirectional replication *via* theta type of replication intermediates. The nature of another replication mechanism utilized by HPV18 involves most likely recombination-dependent replication. In this paper, we show that the usage of different replication mechanisms is a property shared also by other HPV types, namely HPV11 and HPV5. We further show that the emergence of the recombination dependent replication coincides with the oligomerization of the viral genomes and is dependent on the replicative DNA polymerases. We also show that the oligomeric genomes of HPV18 replicate almost exclusively using recombination dependent mechanism, whereas monomeric HPV31 genomes replicate bi-directionally during the maintenance phase of the viral life-cycle.

## Introduction

Papillomaviruses are small DNA viruses that infect the cutaneous and mucosal epithelium. Persistent infection of a number of different human papillomavirus (HPV) types can lead to various types of malignancies (Graham, 2017). A unique feature of the HPV life-cycle, shared by Epstein-Barr Virus, is that the viral genome is maintained in the infected cells as an extrachromosomal nuclear replicon over a long period of time. The HPV infection-cycle can be divided into three distinct steps based on the behavior of the viral genome in the cell. First, initially following the infection the copy-number of the viral genome increases as it replicates more than once in the cell-cycle. Second, during the latent infection, the copy number of the viral genome is maintained constant over time, and the virus replicates in concert with the host cell genome. Third, the viral copy number increases again, and new virus particles are formed. The latter takes place only in the terminally differentiated keratinocytes (McBride, 2017).

The HPV genomes are replicating using largely the host cell replication machinery. The only viral elements necessary for the HPV replication during the initial amplification stage of the infection cycle, are two viral proteins E1 and E2, and an origin of replication (ori) residing in the non-coding part of the viral genome (McBride, 2017). The viral proteins are necessary for the recognition of the ori and recruitment of the host cell replication machinery to it. In addition, the E1 protein possesses helicase activity and is believed to be present in the replication elongation complex (Bergvall et al., 2013).

It is unclear, however, how the latent phase of the HPV life-cycle is established, why the replication of viral genomes becomes limited to one time per cell cycle, and which viral elements are necessary during this stage of the viral infection. Several indications suggest that the mechanism and determinants of the viral genome replication might differ during the latent infection as compared to that during the initial amplification. First, it has been shown that while the initial amplification of the HPV11 and HPV18 genomes proceeds bi-directionally, *via* theta type of replication intermediates, starting from the viral ori, later another, unknown replication mechanism (referred here as non-theta) gradually replaces it, as assessed by the analysis of the replication intermediates using two-dimensional (2D) gel electrophoresis in the U2OS and HaCat cells (Orav et al., 2015, 2019). The maintenance replication of the HPV18 genomes occurs almost exclusively using this non-theta replication mechanism in the U2OS cells (Piirsoo et al., 2020). Second, in contrast to the initial infection, when a monomeric 8kb HPV genome infects the cell, the viral genomes tend to form oligomeric concatemeric structures in the beginning or during the stable replication phase of the viral life-cycle (Frattini et al., 1996; Orav et al., 2013; Warburton et al., 2018; Piirsoo et al., 2020; Lototskaja et al., 2021). Third, it has been shown that the viral protein E1 is dispensable for the maintenance replication of various HPV types (Egawa et al., 2012; Murakami et al., 2019; Piirsoo et al., 2020). In addition, we have previously shown that the maintenance replication of the major oligomeric form of the HPV5 genome is independent of the viral E2 protein in U2OS cells (Lototskaja et al., 2021).

The above-described observations have led us to further analyze the nature of the non-theta HPV replication mechanism in an attempt to understand the mechanism of transition from the initial amplification to the stable maintenance phase of the viral life-cycle. In this paper, we show that the non-theta replication mechanism, previously observed only in the case of mucosal alpha HPV types, is present also in the beta HPV5 genome in the U2OS cells. We also show that the non-theta replication of the HPV genomes is not confined to the artificial cellular systems but takes also place in the CIN612 cells, which are natural host cells for HPV and contain stably replicating HPV31 genomes. Finally, we establish that the appearance of non-theta mechanism in the HPV replication is connected with the oligomerization of the viral genomes.

## Materials and Methods

### DNA Constructs

Plasmids containing HPV5, HPV11, HPV18, HPV18E, and HPV18E8-genomes on the basis of the pMC.BESBX minicircle production vector have been described previously (Sankovski et al., 2014; Orav et al., 2015). Minicircle genomes were generated in *Escherichia coli* strain ZYCY10P3S2T using the minicircle DNA technology as previously described (Orav et al., 2015). Plasmids pUCURR18 and pGLURR18 harboring HPV18 URR, and HPV18 E1 (pMH18E1), and E2 (pQM18E2) expression vectors have been described previously (Kadaja et al., 2007; Kurg et al., 2010). pGEXURR18 was constructed by digesting the pGEXGSTFar1 plasmid (kind gift from Dr. Mart Loog) with XbaI to remove the yeast Far1 cDNA, blunting the 5' overhangs with Klenow fragment and cloning the blunted HPV18 URR BamHI fragment (nt 6929-119) into it.

### Cell Culture and Transfections

Human osteosarcoma cell line U2OS (ATCC No HTB-96) and SV40 transformed African green monkey kidney cell line Cos1 (ATCC No CRL-1650) were propagated in the normal growth medium containing Iscove’s Modified Dulbecco’s Medium, 10% fetal calf serum (FCS) and 1% penicillin/streptomycin at 37°C, and 5% CO2. The cells were transfected by electroporation (975μF and 220V for U2OS or 180V for Cos1 and) using a Gene Pulser XCell system (Bio-Rad Laboratories). The following amounts of wt or modified HPV minicircle genomes were used for the transfection of 2×106 U2OS cells: HPV5 and HPV18E 2μg and HPV11 and HPV18E8-0.5μg. A total of 2μg of HPV18 URR containing plasmids (pUCURR18, pGLURR18, or pGEXURR18) were transfected together with 50μg of pMH18E1 and 250ng of pQM18E2 expression vectors to 2×106 U2OS cells. A total of 0.5μg of pCDNA3 was transfected to 2×106 Cos1 cells.

CIN612 cells (kind gift from Dr. Frank Stubenrauch) were grown in the Defined Keratinocyte-SFM Medium (DKSM; Gibco, Thermo Fisher Scientific). Cells were detached using 0.05% Trypsin-EDTA solution, immediately transferred into DKSM containing 2.5% FCS, centrifuged at room temperature (RT) 1200rpm for 5min, and plated onto a new culture dish and fresh DKS after every 3–4days. The cells were allowed to differentiate in the DKSM containing 1.5mM Ca++ for 72h.

### 1D and 2D Analysis of Replication

Episomal or genomic DNA was isolated from cells as described previously (Geimanen et al., 2011). DNA isolated from the transient replication assays was digested with a restriction endonuclease DpnI, to eliminate input, non-replicated DNA. The following enzymes were used to linearize replicons analyzed: BglI (HPV18, HPV18E, and HPV18E8-), SacI (HPV5), BstEII (HPV11), BstXI (HPV31), XbaI (pUCURR18, pGLURR18), NcoI (pGEXURR18), and XmaI (pCDNA3). The following enzymes were used as noncutters for the replicons analyzed: HindIII (HPV18), BamHI (HPV31), BglII (pUCURR18), EcoRI (pGLURR18), and KpnI (pGEXURR18).

One dimensional (1D) and 2D analysis of replication by Southern blotting (SB) was performed as previously described (Orav et al., 2015). A total of 3–5μg-s of DNA was used for 1D analysis of replication, 120μg (HPV18E8- and pCDNA3), or 150μg (all other replicons) of the episomal DNA was used for 2D analysis of replication intermediates.

Signals from Southern blots were manually marked and pixels in the marked areas were counted using ImageQuant software. Background noise was separately substracted from every blot. Data was analyzed using Graphpad software.

### Statistical Analysis

The values of *p* for the two-tailed *t*-test were based on assumed equal variances and calculated using Excel software.

### Cell Cycle Analysis

Cell cycle profiles of U2OS cells were analyzed using propidium iodide staining and FACS analysis as described previously (Piirsoo et al., 2019).

### Immunoblotting

Western blot analysis was performed as previously described (Kauts et al., 2013). The E1 protein was detected using mouse anti-HA antibody (clone HA-7, Sigma-Aldrich) at a dilution 1:3,000. The E2 protein was detected using mouse monoclonal antibody 2E7.1 against HPV18 E2 protein, at a dilution 1: 2,500 (kind gift from Mihkel Allik).

## Results

### Transient Replication of the HPV Genomes Involves Two Distinct Mechanisms

We and others have previously shown that the initial amplification of the genus alpha HPV11 and -18 genomes proceeds *via* at least two distinct mechanisms, with one involving the theta type of replication intermediates (Orav et al., 2015, 2019). These two mechanisms were observed in both U2OS and HaCat cells, indicating that it is not a cell type specific phenomenon. We were therefore interested, if the replication of the genus beta HPV genomes also involves different mechanisms. We used U2OS cells as a cell culture model permissive for HPV genome replication (Geimanen et al., 2011) and compared the replication mechanisms of HPV5 to those of HPV11 and HPV18. It has been previously shown that all these genomes replicate in the U2OS cells, with HPV5 having the lowest and HPV11 having the highest efficiency of replication (Geimanen et al., 2011).

**Figure 1:**
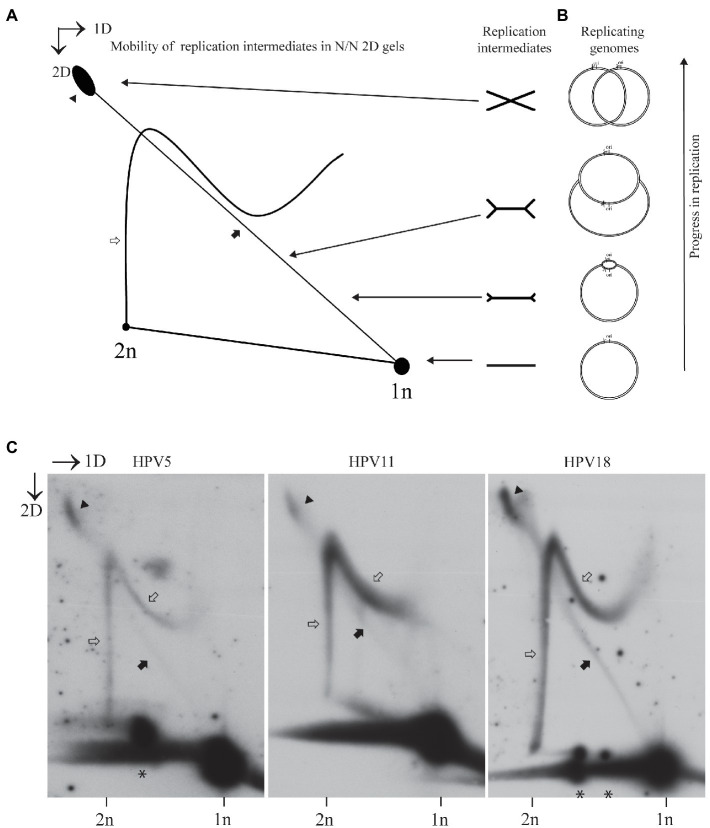
Usage of the non-theta replication mechanism during transient replication is characteristic to different human papillomavirus (HPV) types. **(A)** Schematic representation of the expected mobility of the theta replication intermediates in neutral-neutral two-dimensional (2D) gel in case a circular replicon is linearized near the origin of replication (ori). Almost a straight diagonal line emanating from the spot of 1n linear molecules represents a doubleY structure, with two replication forks that started from the ori to opposite directions advancing as the line retreats from the spot of 1n linear molecules. A non-linear line ending in the 2D spot represents the structure arising from the non-theta type of replication. **(B)** Sketch of the HPV replicon at the different stages of bidirectional replication. Ori represents origin of replication; scissors refer to the position, where the HPV genome is linearized. **(C)** Neutral-neutral 2D analysis of the replication intermediates arising from the HPV5, HPV11, and HPV18 genome replication after transient transfection of the respective genomes into U2OS cells. Episomal DNA was extracted 72h post-transfection, linearized with a restriction enzyme cutting near ori [HPV5 – SacI (nt 702); HPV11- BstEII (nt 7,899) HPV18 – BglI (nt 7,656)] run on an agarose gel at two dimensions and analyzed by Southern blotting (SB). Black arrows refer to the theta replication intermediates, black arrowheads refer to the almost fully replicated genomes, white arrows refer to the non-theta replication intermediates, asterisks depict the linear HPV genome fragments running between the size of 1n and 2n.

Mechanisms of replication were elucidated by analyzing the replication intermediates that can be resolved in 2D electrophoresis, followed by SB analysis of the specific signals. Different types of 2D systems can be used to study the replication mechanisms, and depending on the nature of replicon and site of digest relative to the ori, different patterns of the replication intermediates can be observed (Henno et al., 2017). We chose neutral-neutral 2D system and digested HPV genome once in the vicinity of the replication origin, since the migration pattern of replication intermediates arising under these conditions are easy to interpret and explain. Schematic representation of the expected replication intermediates arising from a bi-directionally replicating circular replicon and analyzed under the above-mentioned conditions are depicted in Figures 1A,B. Majority of the analyzed replicons are not replicating at a given moment, since only a limited number of cells in a population are in the S phase. Non-replicating molecules run as spots of 1n and 2n. 1n spot is always more prominent, since the G1 phase is the longest and has the highest number of cells. The line between 1n and 2n molecules represents linear arc and probably consists of replicating molecules, where replication fork structure has been broken either in the cells or during the extraction of DNA. Major replication intermediates that can be observed using this type of analysis are called as doubleY and simpleY structures. DoubleY structures represent replication intermediates with two replication forks emanating from the ori and proceeding away from each other (called as converging forks). Circular replicons have often trouble finishing replication, and therefore the most abundant portion of the doubleY structures is almost fully replicated molecules. SimpleY structures indicate either unidirectional replication or result from the bidirectional replication, where one of the replication forks has been stalled early on for some reason. It has also been suggested that simpleY structures might represent the rolling circle type of replication in case of SV40 (Sowd et al., 2013).

The HPV5, HPV11, or HPV18 genomes were transfected into the U2OS cells, and episomal DNA was extracted 72h post-transfection. Episomal DNA was digested with a restriction endonuclease cutting the respective genome once near the ori, resolved on two dimensions and hybridized with a probe for the specific HPV type.

As seen in Figure 1C, all three HPV types analyzed had similar replication intermediates. The signal intensity was proportional to the replication efficiency observed previously (Geimanen et al., 2011), as HPV5 replication was the weakest, and HPV11 replication was the strongest. DoubleY structures were observed in each case (black arrows), with substantial amounts of almost fully replicated molecules present (black arrowheads). Accumulation of almost fully replicated molecules indicates that the finishing of the HPV replication is problematic, which is a common problem among circular replicons.

In addition, all analyzed HPV types contained additional non-theta replication intermediates, as previously observed in the case of HPV11 and HPV18 (white arrows). To the best of our knowledge, the migration pattern of these intermediates does not concur with any type of replication intermediates observed in other systems, where the DNA replication mechanisms have been studied using 2D analysis.

The complete lack of the simpleY structures in the case of HPV theta replication should also be mentioned. This is in sharp contrast with the replication of a related DNA virus SV40 and yeast 2-micron plasmid, where simpleY structures showing unidirectional replication are present along with doubleY structures, if a replicon is cleaved once in the vicinity of the ori (Brewer and Fangman, 1987; Sowd et al., 2013). We also often observed discreet spots running on the linear arc of molecules between the lengths of 1n (8kb) and 2n (16kb) molecules (depicted by asterisks in Figure 1C). These molecules represent partially replicated HPV genomes, indicating that replication of HPV genomes is inefficient, and collapse of replication forks is frequent.

**Figure 2:**
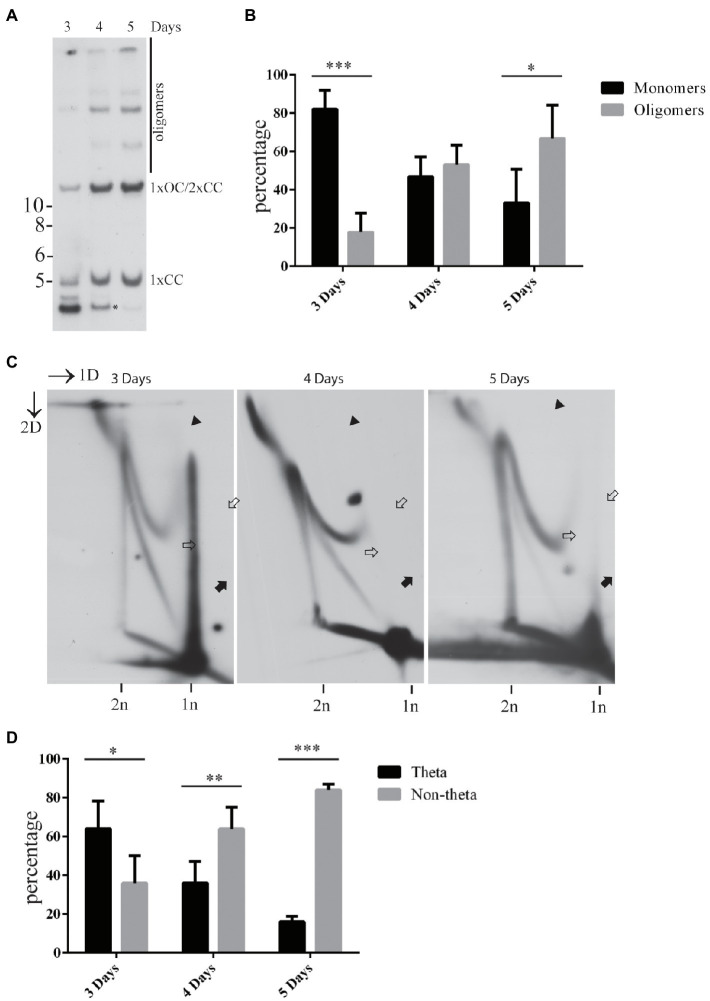
Appearance of the non-theta replication intermediates during the transient replication of the HPV18 genomes in the U2OS cells concurs with the oligomerization of the replicated viral genomes. **(A)** An example of different forms of the HPV18 genome arising as a result of the transient replication in U2OS cells at 3, 4, or 5days after the transfection. Genomic DNA was extracted from the cells at indicated time points, digested with HPV18 non-cutter HindIII and DpnI to separate non-replicated input DNA, resolved on 0.6% gel and analyzed by SB. Migration of the monomeric closed circular (1xCC), monomeric open circular (1XOC), dimeric closed circular (2xCC), and oligomeric forms are shown. Asterisk represents migration of non-replicated, DpnI sensitive fragment of HPV18. **(B)** Quantification of the replicated monomeric and oligomeric HPV18 genomes at 3, 4, and 5days after the transfection in the U2OS cells. The data are expressed as average means±the SD (^*^p<0.05; ^**^p<0.01; and ^***^p<0.001) of four independent experiments. Monomeric open circular and dimeric closed circular genomes migrate together under the conditions used to resolve DNA and were therefore omitted from calculation. **(C)** An example of different types of the replication intermediates arising during the transient replication of the HPV18 genome in U2OS cells at 3, 4, and 5days post-transfection. Black arrows refer to the theta replication intermediates, black arrowheads refer to the almost fully replicated genomes, white arrows refer to the non-theta replication intermediates. **(D)** Quantification of the theta- and non-theta replication intermediates arising during the transient replication of the HPV18 genomes at 3, 4, and 5days after the transfection in the U2OS cells. The data are expressed as average means±the SD (^*^p<0.05; ^**^p<0.01; and ^***^p<0.001) of four independent experiments.

We have previously shown that the abundance of the non-theta replication intermediates increases over time during the initial amplification of the HPV18 genomes in the U2OS cells (Orav et al., 2015). At the same time, the HPV genomes tend to form oligomers, if transfected into the U2OS cells (Orav et al., 2013). We were therefore interested to analyze if the appearance of the oligomeric HPV genomes coincides with the appearance of the non-theta replication intermediates. To do that, we quantified the amounts of the replicated monomeric and oligomeric HPV18 genomes at 3, 4, and 5days after transfection and compared that to the abundance of the theta- and non-theta replication intermediates at the same time points (Figure 2). The monomeric HPV genomes and the theta intermediates were prevalent at 3days post-transfection. Four days after the transfection, the amounts of the monomeric and oligomeric genomes were similar, whereas almost 70% of the replication intermediates were of non-theta type. It should be noted, however, that the amounts of the oligomeric genomes may be underestimated in our quantitation, since it is virtually impossible to separate monomeric open circular and dimeric closed circular genomes under these gel conditions. At 5days post-transfection more than 60% of the replicated HPV18 genomes were oligomeric and more than 80% of the replication intermediates were of non-theta type (Figures 2B,D). Representative images of 1D (used for quantification of the monomeric and oligomeric HPV genomes) and 2D (used for quantification of the theta and non-theta intermediates) SB are shown in Figures 2A,C.

Taken together, these data show that two different replication mechanisms are utilized by different HPV types during the initial amplification of the viral genomes in the U2OS cells. Appearance of the non-theta intermediates over time indicates their relation with the formation of the oligomeric HPV genomes. In addition, the accumulation of almost fully replicated genomes in the doubleY arc and partially replicated genomes in the linear arc indicates that the HPV replication is inefficient in both elongation and termination (or partitioning) phases.

### Appearance of the Non-theta Replication Intermediates Is Dependent on the Activity of Replicative DNA Polymerases

One possible explanation for the non-theta type of intermediates observed during the HPV replication is that these structures represent recombination, rather than replication intermediates. It has been shown in yeast that Holliday junctions, as intermediates of the homologous recombination, can occur at least partially independently of the replication (Zou and Rothstein, 1997). It is widely established that the HPV infection induce ATM and ATR mediated DDR response in the host cell and the virus utilizes homologous recombination mediated replication, although the exact molecular details of this process are poorly understood (Sakakibara et al., 2013). Moreover, it has been shown that the HPV genomes tend to form oligomeric structures *via* homologous recombination in the U2OS cells (Orav et al., 2013).

If the formation of the recombining structures is not dependent on the DNA polymerases, inhibiting DNA polymerase activity should not prevent their formation. We tested this possibility by challenging U2OS cells transfected with the HPV18 genome with a reversible inhibitor of replicative DNA polymerases, aphidicolin.

**Figure 3:**
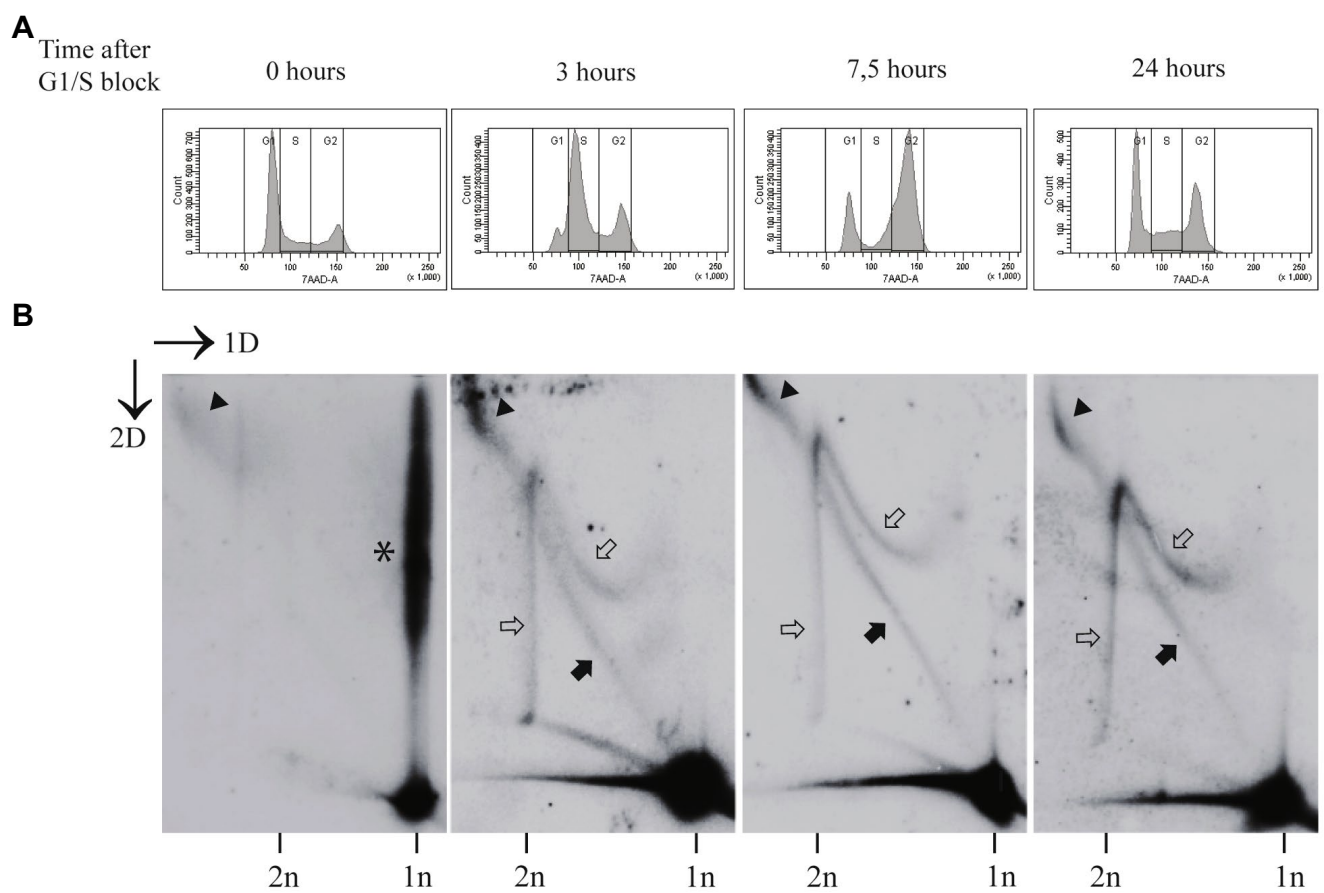
The non-theta replication of the HPV18 genomes is dependent on the replicative DNA polymerase(s). **(A)** The cell cycle profile of the aphidicolin arrested cells (0h), followed by the release into the cell cycle at various time points (3, 7.5, and 24h) post-arrest. The U2OS cells were transfected with the HPV18 genome, 48h after the transfection the cells were arrested at G1/S phase using aphidicolin treatment (2μg/ml) for 24h, followed by the removal of the drug and release of the cells to cycling conditions for the indicated periods of time. **(B)** Neutral-neutral 2D analysis of the replication intermediates arising from the HPV18 genome replication after the transfection into the U2OS cells at various time points after the G1/S block using aphidicolin. Nearly all replication intermediates are absent at the end of 24-h block by aphidicolin (0h). Instead, the collapsed intermediates migrate above the 1n linear molecule spot (indicated by asterisk). At 3h post-release, when majority of the cells have entered the S-phase, both theta and non-theta type of replication intermediates are present. About 7.5h after the release majority of the cells have entered G2/M phase, and still both type of replication intermediates are detectable. At 24h post-release, the cells have restored the normal distribution of the cell cycle and both theta- and non-theta replication intermediates can be seen. Black arrows refer to the theta replication intermediates, black arrowheads refer to the almost fully replicated genomes, and white arrows refer to the non-theta replication intermediates.

Exposure of the U2OS cells to aphidicolin for 24h, starting the treatment 48h after the transfection, induced substantial cell cycle arrest, with 62% of the cells residing in G1, 14% in S and 24% in the G2/M phase (Figure 3A). As expected, this arrest was reversed, when aphidicolin was removed. Within 3h in the normal growth medium, the cell cycle profile was changed dramatically, and cells entered successfully into the S phase (36% in G1, 39% in S, and 25% in G2/M). At 7.5h after the release into the cell cycle, majority of the cells had reached G2/M phase of the cell cycle (21% in G1, 24% in S, and 55% in G2/M). After 24h the normal distribution of the cell cycle was recovered (44% in G1, 25% in S, and 32% in G2/M; Figure 3A).

Two-dimensional analysis of the HPV18 genome replication showed that almost all recognizable replication intermediates were missing in the cells challenged with aphidicolin, with only small amounts of almost fully replicated molecules present (black arrowhead; Figure 3B, left panel). Majority of the HPV molecules were retained in the 1n spot or migrated as branched structures above 1n spot (asterisk in Figure 3A). We believe that these molecules represent the open structures with replication forks stalled due to the DNA polymerase inhibition. At 3, 7.5, and 24h after the release of the cells into the cell cycle, all types of HPV18 replication intermediates reappeared (Figure 3B).

These data indicate that replicative DNA polymerase(s) are involved in the formation of the non-theta replication intermediates.

### Mechanism of Replication Initiated From the HPV18 Ori Depends on the Size of the Replicon and Not the Levels of the Viral Replication Proteins E1 and E2

One possible prerequisite for the non-theta type of replication is the virus specific transcriptional regulation, which results in the production of correct levels of the viral replication proteins E1 and E2. These levels can be altered by co-transfecting the viral genome with the expression vectors coding for E1 and E2.

**Figure 4:**
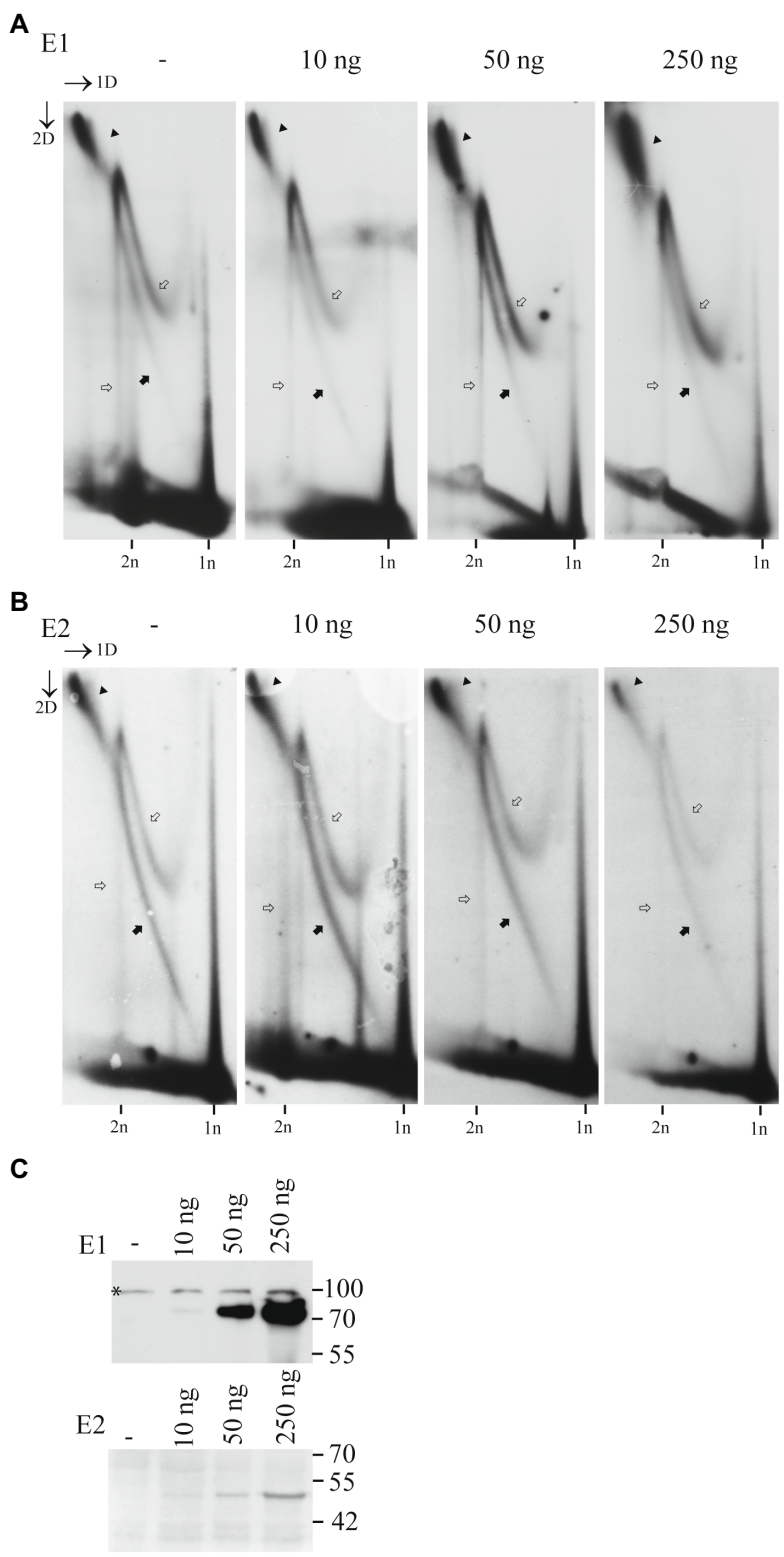
Mechanism of replication initiated from the HPV18 ori does not depend on the E1 and E2 protein levels. **(A)** Neutral-neutral 2D analysis of the replication intermediates arising from the HPV18 E8-genome replication in the presence of growing amounts of the E1 expression vector. **(B)** Neutral-neutral 2D analysis of the replication intermediates arising from the HPV18 E8-genome replication in the presence of growing amounts of the E2 expression vector. Episomal DNA was extracted 72h after the transfection, linearized with a restriction enzyme BglII cutting near the ori, run on an agarose gel at two dimensions and analyzed by SB. Black arrows refer to the theta replication intermediates, black arrowheads refer to the almost fully replicated genomes, white arrows refer to the non-theta replication intermediates. **(C)** Western blot analysis showing the expression levels of the HPV18 E1 and E2 proteins, when co-transfected together with the HPV18 E8-genome.

To test, if the levels of E1 and E2 could alter the replication mechanism of HPV18, we used mutant HPV18 genome, which does not express E8/E2 repressor protein, and therefore replicates better than the wt HPV18 (Reinson et al., 2013). We co-transfected the mutant genome together with increasing amounts of the HPV18 E1 or E2 expression vectors into the U2OS cells and analyzed the mechanisms of replication by 2D gel electrophoresis followed by SB. Results of this analysis are shown in the Figure 4. We could not detect any major changes in the relative distribution of different types of replication intermediates even at high levels of E1 or E2 present. Therefore, we can conclude that the usage of distinct type of replication mechanisms is not dependent on the levels of the viral replication proteins E1 and E2.

These data indicate that the formation of the non-theta intermediates is dependent on the replicative DNA polymerase(s), and they represent *bona fide* replication intermediates.

Replication from the HPV ori can proceed also, if the origin of replication is cloned into a heterologous plasmid and the viral proteins E1 and E2 are provided in trans from the expression vectors. We used this set-up to analyze, if any other viral protein or cis-elements lying outside the viral ori, might influence the mechanism of HPV replication. We used replicons of different sizes containing HPV18 URR (nt 6929-119). This 1.1kb fragment contains all the necessary cis elements for replication (Remm et al., 1992). This fragment was cloned into pUC (pUCURR18), resulting in a 4-kb replicon, pGL4 (pGL4URR18), resulting in a 6.5-kb replicon, and into pGEXFar1 (pGEXFarURR18), resulting in a 8.2-kb replicon. The plasmids were transfected into the U2OS cells together with the HPV18 E1 and E2 expression vectors, and their replication was analyzed 72h after the transfection. For the analysis of the replication, we extracted episomal DNA and digested it with the restriction endonucelases that either linearize the replicon or do not cut the replicon. All digestions were performed in the presence of DpnI enzyme to cut transfected, non-replicated DNA and expression vectors.

**Figure 5:**
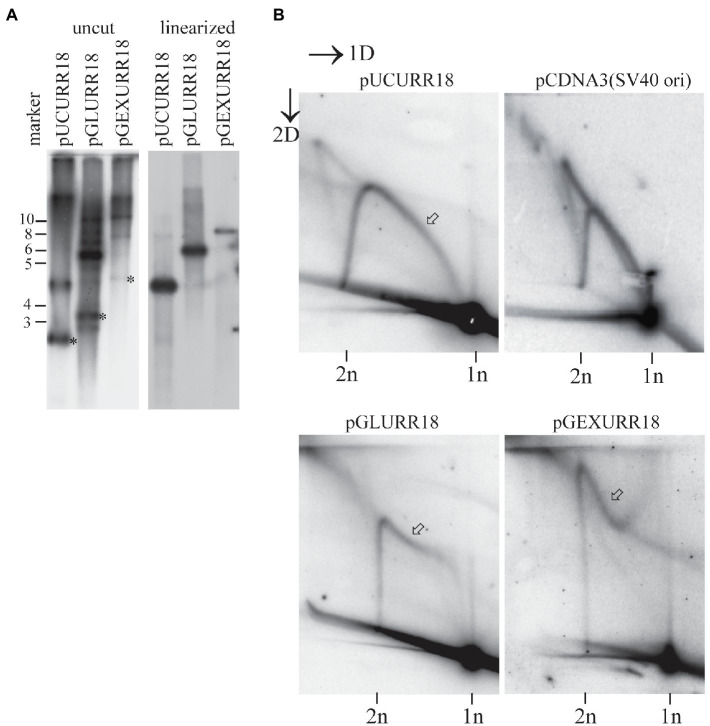
Mechanism of replication initiated from the HPV18 ori depends on the size of the replicon. **(A)** Transient replication of the HPV18 ori harboring replicons with different sizes in the U2OS cells. pUCURR18 (4kb), pGLURR18 (6kb), or pGEXURR18 (8,2 kb) were transfected together with the HPV18 E1 and E2 expression vectors into the U2OS cells. Episomal DNA was extracted 72h after the transfection, digested with replicon non-cutter (left panel) or single cutter enzyme (right panel), combined with DpnI to digest the non-replicated plasmids, resolved in 0.8% agarose gel and analyzed by SB. Asterisks represent migration of the monomeric closed circular forms of the respective replicons. **(B)** Neutral-neutral 2D analysis of the replication intermediates arising from the HPV18 harboring replicons of different sizes during the transient replication in the U2OS cells and the SV40 origin containing pCDNA3 replicon in Cos1 cells. The replication intermediate structure that varies between different HPV replicons is indicated with an arrow.

Southern blotting analysis showed that all three constructs were able to replicate in the U2OS cells given that the HPV18 E1 and E2 expression vectors were co-transfected (Figure 5A). Analysis of the linearized replicons showed that all replicated plasmids were of the expected sizes (Figure 5A, right panel). Analysis on the noncut replicons showed that all plasmids formed oligomeric structures in the cell (Figure 5A, left panel).

Two-dimensional analysis of the respective replicons was performed by linearizing the replicons once near the HPV18 ori. The analysis showed that the 4kb pUCURR18 plasmid replicated strictly *via* theta type of intermediates, with substantial amounts of singleY structures present, indicating unidirectional replication (Figure 5B, left panel). This distribution of the replication intermediates was almost indistinguishable from the replication of SV40 origin containing 5.4kb pcDNA3 plasmid in Cos1 cells (Figure 5B, upper panel).

The pattern of the replication intermediates was different in the case of 6.5kb pGLURR18 and 8.2kb pGEXURR18 replicons (Figure 5B, lower panel). Both replicons had the non-theta replication as a prevailing mechanism, while the start of the non-theta intermediate differed between them. By start, we mean the tail of the intermediate that migrates at the size of 1n molecules. The start of the 6.5kb pGLURR18 replicon was directed toward linear 1n molecules, while the start of 8.2kb pGEXURR18 molecules was directed away from it.

These data show that the non-theta replication of the HPV genomes is not dependent on the E1 and E2 levels, or any cis element lying in the coding region of the genome, but rather is a function of the replicon size.

### Maintenance Replication of the HPV31 Genomes in CIN612 Keratinocytes Proceeds *via* Two Distinct Mechanisms

**Figure 6:**
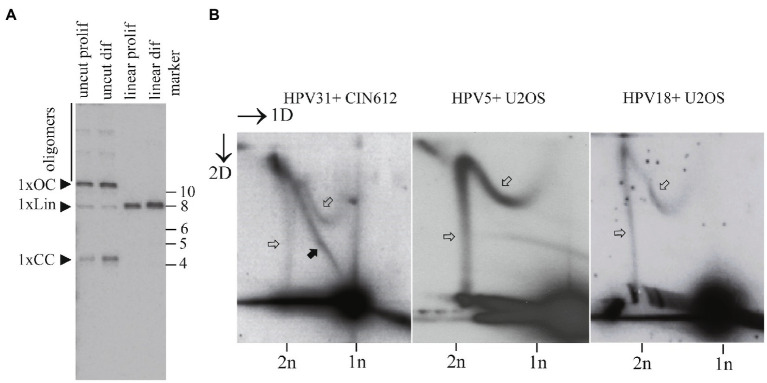
Maintenance replication of the HPV31 genomes proceeds *via* distinct mechanisms in the CIN612 keratinocytes. **(A)** SB showing the status of the HPV31 genomes in CIN612 cells. HPV31 noncutter (BamHI) or single cutter (BstXI) enzymes were used to digest episomal DNA. Migration of the monomeric closed circular (1xCC), monomeric open circular (1XOC), linear (1xLin) and oligomeric forms are shown. **(B)** Neutral-neutral 2D analysis of the replication intermediates arising from the HPV31 maintenance replication in the CIN216 cells, HPV5 and HPV18 maintenance replication in the U2OS cells. Black arrows refer to the theta replication intermediates; white arrows refer to the non-theta replication intermediates.

In order to corroborate our results about distinct replication mechanisms utilized by the HPV genomes obtained from U2OS based cell systems, we used a different cell type, CIN612 keratinocytes, which is permissive for HPV replication. These cells harbor stably replicating HPV31 genomes as extrachromosomal plasmids. In sharp contrast to the U2OS based stable HPV positive cell lines, majority of HPV genomes are monomeric in CIN612 cells (Figure 6A).

Analysis of the replication mechanisms utilized by HPV31 genomes in CIN612 cells showed that a dominating mechanism of replication is that of theta type (Figure 6B, black arrows). However, we could also detect the non-theta replication intermediates that are similar to those observed in U2OS cells for other HPV types (Figure 6B, white arrows).

As a comparison, we used HPV5+ and HPV18+ U2OS cells (previously referred as clone 15 and clone 1.13 in Geimanen et al., 2011). These cells have oligomeric HPV genomes stably replicating in the cells (Geimanen et al., 2011; Piirsoo et al., 2020). As shown in the Figure 6B, these oligomeric genomes replicate exclusively using the non-theta type of the replication intermediates.

The low abundance of the non-theta type of replication in the CIN612 cells coincides with the amounts of oligomeric forms of the HPV31 genomes in these cells.

## Discussion

DNA replication in the mammalian cell nuclei can take place *via* at least three different mechanisms. The most widely used mechanism utilizes the classical semiconservative synthesis of new DNA strands starting from specific sequences, called as origins of replication. This type of replication is essential for the maintenance of the genome integrity, and it has been believed for a long time that the HPV genomes are also replicated this way. Second mechanism of nuclear DNA replication involves break induced replication (BIR), that is activated upon stress conditions to repair the double strand breaks and collapsed classical replication forks (Malkova, 2018). This type of DNA replication does not need specific start sites and leads to the genomic rearrangements. Yet another type of replication, that also does not start from the specific origins, called as rolling circle replication, can take place in the mammalian cell nuclei (Wawrzyniak et al., 2017). This replication mode is utilized by some DNA viruses, including adeno-associated virus and human herpesvirus 6, and results in the concatemeric multimers of the initial replicon (Tattersall and Ward, 1976; Astell et al., 1985; Borenstein and Frenkel, 2009).

All these above-mentioned replication mechanisms have been implicated to be operating during the HPV replication. Semiconservative bidirectional replication has been shown to be the HPV replication mechanism using the analysis of the replication intermediates in 2D gels in the case of HPV11, HPV16, and HPV31 in laryngeal papillomas, W12 and CIN612 cell lines, respectively (Auborn et al., 1994; Flores and Lambert, 1997). Rolling circle type of HPV16 and HPV31 replication has been proposed to take place upon keratinocyte differentiation and could therefore represent the replication mode in the last, vegetative phase of the viral life cycle (Flores and Lambert, 1997). However, these studies actually analyzed subgenomic fragments the whole HPV replicon, which could affect the interpretation of the results. We have shown previously that when the whole HPV18 replicons, extracted from transiently transfected U2OS cells, are investigated using the analysis of the replication intermediates in 2D gels, mixed type of replication could be observed (Orav et al., 2015). Initially most of the HPV18 replication proceeds *via* bidirectional mechanism, but later it is gradually replaced by another mode, which we believe to be BIR. It has been shown that expression of HPV oncoproteins induce replication stress, which in turn activate BIR (Malkova, 2018; Moody, 2019). In the present article, we extend these findings and show that other HPV types, HPV5 and HPV11, also utilize similar mixed replication mechanism upon the initial amplification in the U2OS cells. We also show that HPV31 utilizes RDR during maintenance replication in keratinocytes, similarly to the maintenance replication of HPV18 in the U2OS derived cell-lines (Piirsoo et al., 2020).

One of the questions we have tried to answer in this article is what distinguishes the HPV18 genomes replicating in a bidirectional manner vs. those replicating *via* BIR. It has been previously shown, that the HPV genomes form oligomeric structures through homologous recombination (Orav et al., 2013). We observed that BIR type of replication starts to occur only after the oligomeric HPV18 genomes appear in the cells. Furthermore, the abundance of BIR is proportional to the abundance of the oligomeric HPV genomes in the transiently transfected U2OS cells, and in the stable cell lines carrying episomal HPV31 genomes. Therefore, we hypothesize that while monomeric HPV genomes replicate bi-directionally in an E1 and E2 dependent manner, oligomeric HPV genomes replicate using BIR that does not require viral trans factors. Further support to this hypothesis comes from the studies showing that viral the E1 protein is dispensable for the maintenance replication of the HPV genomes (Hubert and Laimins, 2002; Fradet-Turcotte et al., 2010; Egawa et al., 2012; Murakami et al., 2019). We have also recently shown that the maintenance replication of the major topological forms of HPV18 genomes in the U2OS cells is independent of the E1 function (Piirsoo et al., 2020). Furthermore, the major topological forms of the HPV5 genomes do not require the function of E2 protein during the maintenance replication in the U2OS cells (Lototskaja et al., 2021).

One of the possibilities, why the bidirectional HPV replication is replaced by BIR, is poor expression of E1 and E2 from the viral genomes, so that the viral origins of replication are insufficiently fired. To test this possibility, we investigated if the balance between bidirectional replication mode and BIR can be altered by forced expression of the E1 or E2 proteins from the expression vectors. However, we found that the over-expression of neither E1 nor E2 could change this balance toward the bidirectional replication mode. Instead, we found that the distance between two HPV replication oris could alter the replication mechanism. Oligomeric concatemer forms of the HPV genomes have the spacing between the two oris of around 8kb. These molecules replicate using BIR. When an 8-kb plasmid containing HPV18 URR was transfected into the U2OS cells together with E1 and E2 expression vector, it also formed oligomeric structures and replicated using BIR. However, when a 4-kb plasmid with HPV18 URR was used in a similar experiment, it formed oligomers but continued to replicate in a bidirectional manner. These data suggest that smaller spacing between HPV oris could allow the oligomeric molecules to replicate in a bidirectional manner. The reasons why spacing between the HPV origins of replication is important in determining the replication mode remains to be investigated. One possibility is that the HPV E1 protein, the only viral factor present in the replication elongation complex, is not a very efficient helicase and causes stalling and collapse of the replication forks, which in turn creates substrates for BIR replication. With shorter distances between two oris, however, there is higher chance of completing the bidirectional replication.

Taken together, we propose that a general course of the HPV infection involves the initial E1 and E2 dependent amplification of the viral genomes after the infection, which is accompanied by the replication stress. The replication stress is induced by the expression of HPV oncogenes E6 and E7 (Moody and Laimins, 2009; Moody, 2019) and the inability to fully duplicate the viral genomes using bi-directional replication mechanism. The replication stress leads to the oligomerization of the viral genomes, which start to replicate using the BIR dependent mechanism.

## Data Availability Statement

The raw data supporting the conclusions of this article will be made available by the authors, without undue reservation.

## Author Contributions

MP and MU designed the experiments. MP, AP, MU, and EU guided the work. LL, AP, AnL, E-MT, and AiL performed the experiments. MP and AP wrote the manuscript. LL, AP, AnL, E-MT, AiL, EU, MU, and MP edited the manuscript. All authors contributed to the article and approved the submitted version.

## Funding

This research was funded by the European Regional Development Fund through the Centre of Excellence in Molecular Cell Engineering, Estonia (2014-2020.4.01.15-013), an institutional research funding grant (IUT20-27) from the Estonian Research Council, and a personal research funding team grant (PRG198; awarded to MU) from the Estonian Research Council. The funders had no role in study design, data collection and analysis, decision to publish, or preparation of the manuscript.

## Conflict of Interest

The authors declare that the research was conducted in the absence of any commercial or financial relationships that could be construed as a potential conflict of interest.

## Publisher’s Note

All claims expressed in this article are solely those of the authors and do not necessarily represent those of their affiliated organizations, or those of the publisher, the editors and the reviewers. Any product that may be evaluated in this article, or claim that may be made by its manufacturer, is not guaranteed or endorsed by the publisher.
